# Adverse drug reactions in paediatric surgery: prospective study on frequency and risk related factors

**DOI:** 10.1186/s12887-024-04803-1

**Published:** 2024-05-18

**Authors:** C Pérez-Ingidua, AB Rivas-Paterna, C González-Perrino, E Aleo-Luján, A Ascaso-del-Rio, L Laredo-Velasco, A Portolés-Pérez, E Vargas-Castrillón

**Affiliations:** 1 Clinical Pharmacology Department, Hospital Clínico San Carlos, Madrid, Spain; 2 Anaesthesiology and Resuscitation Department, Hospital Clínico San Carlos, Madrid, Spain; 3 Paediatric Intensive Care Unit and Postanaesthesia Recovery Unit, Hospital Clínico San Carlos, Madrid, Spain; 4 Faculty of Nursing, Physiotherapy and Podiatry, Universidad Complutense de Madrid, Madrid, Spain; 5 Faculty of Medicine, Universidad Complutense de Madrid, Madrid, Spain; 6 Instituto de Investigación Sanitaria del Hospital Clínico San Carlos (IdISSC), Madrid, Spain

**Keywords:** Adverse drug reactions, Off-label, Paediatrics, Surgery, Anaesthetics, Causality concordance

## Abstract

**Background:**

Paediatric patients are especially prone to experiencing adverse drug reactions (ADRs), and the surgical environment gathers many conditions for such reactions to occur. Additionally, little information exists in the literature on ADRs in the paediatric surgical population. We aimed to quantify the ADR frequency in this population, and to investigate the characteristics and risk factors associated with ADR development.

**Methods:**

A prospective observational study was conducted in a cohort of 311 paediatric patients, aged 1–16 years, admitted for surgery at a tertiary referral hospital in Spain (2019–2021). Incidence rates were used to assess ADR frequency. Odds ratios (ORs) were calculated to evaluate the influence of potential risk factors on ADR development.

**Results:**

Distinct ADRs (103) were detected in 80 patients (25.7%). The most frequent being hypotension (*N* = 32; 35%), nausea (*N* = 16; 15.5%), and emergence delirium (*N* = 16; 15.5%). Most ADRs occurred because of drug-drug interactions. The combination of sevoflurane and fentanyl was responsible for most of these events (*N* = 32; 31.1%). The variable most robustly associated to ADR development, was the number of off-label drugs prescribed per patient (OR = 2.99; 95% CI 1.73 to 5.16), followed by the number of drugs prescribed per patient (OR = 1.26, 95% CI 1.13 to 1.41), and older age (OR = 1.26, 95% CI 1.07 to 1.49). The severity of ADRs was assessed according to the criteria of Venulet and the Spanish Pharmacovigilance System. According to both methods, only four ADRs (3.9%) were considered serious.

**Conclusions:**

ADRs have a high incidence rate in the paediatric surgical population. The off-label use of drugs is a key risk factor for ADRs development.

**Supplementary Information:**

The online version contains supplementary material available at 10.1186/s12887-024-04803-1.

## Introduction

There are various difficulties surrounding pharmaceutical paediatric research, including the ethical-legal implications of early phase trials in the paediatric population, low industrial interest in small market population studies, and the lack of parental consent for infant participation. This has resulted in an information gap surrounding the safety and efficacy of medicines in this population [1]. Most medicines (up to 90% of all prescriptions) are therefore used off-label [2–4] for paediatric patients. The confluence of these factors, and the physiological differences between children and adults, makes the former particularly susceptible to adverse drug reactions (ADRs) [5, 6].

The surgical environment is ideal for the development of medicine-related problems due to the intake of multiple concomitant drugs and complex pharmacokinetic/pharmacodynamic properties. However, the impact and magnitude of ADRs in the paediatric surgical setting have not been studied sufficiently. Most publications focus on critical events related to anaesthetics [7–11] or hypersensitivity/anaphylactic reactions [12–14], leaving aside ADRs of any other aetiology.

To improve healthcare for the paediatric population, the objectives of this study were to quantify the frequency of ADRs in paediatric patients admitted for surgery, and to assess the characteristics and risk factors associated with ADR development.

## Matherials and methods

### Patients and study design

A prospective cohort study was conducted between august 2019 and October 2021 on paediatric patients admitted for surgery at Hospital Clínico San Carlos, a prestigious tertiary hospital placed in Madrid (Spain), that serves as a center for specialized care with over 5000 professionals from different medical specialties, emergency units, operating rooms, and state-of-the-art diagnostic equipment.

To dilute the confounding factors related to the organizational issues of surgery, like the scheduling of interventions by specialty type each day, the subjects were included sequentially on a different day each week during the study period. All children aged 1–16 years undergoing surgery or examination under anaesthesia on these study days, and whose parents provided written consent, were included in the study.

### Procedure, variables, and follow-up

At the time of admission, demographic characteristics (age, sex, and weight), the patient’s chronic treatment and the medical history that could be related to the potential development of ADRs (previous ADRs, physiological and genetic predisposing factors) were recorded. Additionally, until the patient’s discharge, an intensive pharmacological monitoring protocol was applied. This protocol was based on patient observation, a review of the medical records, and interviews with those responsible for the patients.

All the professionals involved in the development of the study (Anaesthesiologists, paediatric intensivists, pharmacologists, and nursing staff) were previously trained in the ADR intensive monitoring method developed by the Boston Collaborative Drug Surveillance Program [15], as well as, in the definition of ADR taken as a reference in the study with the intention of mitigating possible interobserver variability in their identification. For this purpose, two training sessions were conducted, attended by the entire research team: One before starting the study and another halfway through the study period.

The following data were collected for all prescribed drugs: active substance name, assigned code in the anatomical-clinical classification (ATC), route of administration, dosage, regimen, and treatment duration.

Finally, at the time of discharge, the minors and/or their parents were informed about the phone number to inform us about Adverse Events (AEs) or changes in health status, to identify potential late ADRs.

Based on the definition of ADR from the World Health Organization (WHO), we considered that any change in the health status of the patient (signs, symptoms, or laboratory data) with a potential temporal relationship to any drug administered as a suspected ADR (medication errors are excluded from this definition). Suspected ADRs were evaluated by a committee of specialists in clinical pharmacology, paediatric intensive care, anaesthesia, and registered nurses trained in pharmacovigilance. In cases where the committee confirmed ADR suspicion, the following elements were evaluated: causality, severity, avoidability, mechanism of ADR development, duration of the episode, actions taken after ADR identification, and patient outcomes.

Causality was evaluated according to the Naranjo et al. method [16] and Karch-Lasagna [17] algorithm. The lack of a specific algorithm to evaluate causality in paediatric populations or surgical patients justifies the selection of these well-known methods. Both methods consist of a questionnaire-based score allowing the evaluation of ADR aspects such as previous bibliography references, temporal sequence between ADR onset and treatment administration, and the effect of drug re-exposure or withdrawal. The questionnaires applied the following scoring system categories: doubtful (0), possible (1–4), probable (5–8), and definite (≥ 9) [Naranjo et al.]; or unlikely (≤ 0), conditional (1–3), possible (4–5), probable (6–7), and defined (≥ 8) [Karch-Lasagna]. As several drugs were administered concomitantly during surgery, the ADR was attributed to the drug that produced the highest score in the causality assessment. Similarly, when two or more drugs had the same score, the ADR was attributed to the combination. Suspected ADRs categorized as “doubtful” were removed from the analysis, as a potential bias in the estimation of ADR incidence. The concordance between the results obtained from the two algorithms was evaluated.

Severity was evaluated using two different methods, according to the criteria of Venulet [18] and the Spanish Pharmacovigilance System for Medicinal Products for Human Use. In accordance with Venulet, ADRs were categorized as mild (ADRs did not complicate underlying pathology, no treatment was required, or drug discontinuation was unnecessary), moderate (clear signs and symptoms observed without the involvement of vital organs), or severe (vital risk of death, reduced life expectancy, dysfunction of a vital organ, or an ADR duration longer than one month). Based on the Spanish Pharmacovigilance System criteria, ADRs were classified as non-serious or serious (ADRs that are fatal, life-threatening, require hospitalization, prolong hospital stay, produce persistent disability or incapacity, cause congenital anomalies, or generate a medically significant illness).

ADRs’ avoidability was evaluated according to the Hallas’ et al. criteria [19]. This method classifies ADRs in: Definitely avoidable, possibly avoidable, unavoidable, and not assessable. The allocation to any of the four categories is based on the adequate knowledge of the medical practice, the existence of more appropriate therapeutic alternatives and on the knowledge of the patient’s pharmacological medical history.

Regarding the possible mechanisms behind ADRs, these were classified according to the order proposed by Rawlins and Thomson [20]: Type A (explained by the drug’s mechanism of action, with a clear dose-dependent relationship) and Type B (idiosyncratic reactions, not related to the drug’s mechanism of action).

To evaluate the possible risk factors associated with ADR development, the following variables were selected: age, sex, weight, length of stay, number of drugs prescribed per patient, and l number of off-label drugs prescribed per patient.

All data related to the participants’ characteristics: surgical intervention, administered drugs, and suspicion of ADRs, were recorded on two forms designed by the research team ad hoc using the web-based electronic data capture software REDCap® (Vanderbilt University). Data entry was reviewed by the principal investigator. A collaboration between parents and healthcare professionals was essential to gather sufficient information.

### Statistical analyses

Considering an incidence of ADRs of 17% observed in a study previously conducted in our hospital [21], performed in neonatal population a confidence interval of 95% (95% CI), a precision of ± 5% units, and a replacement rate of 30%, a sample size of 310 infants was required to obtain statistically relevant conclusions. This was calculated using the GRANMO sample size calculation program.

The frequency distribution of the qualitative variables is presented with a 95% CI. Quantitative variables are expressed as mean ± standard deviation (SD), and in the case of very large dispersion, the median is also provided.

Bivariate comparative analysis between patients, with and without ADRs, was performed using the χ2 test for qualitative variables. The comparison of means for quantitative variables was performed using the Student’s t-test (having previously checked their normality by the Kolmogorov-Smirnov test). Data were analysed using the Statistical Package for the Social Sciences (SPSS) r 20.0, and the results were considered significant if *p* ≤ 0.05. The concordance between the results obtained by the two causality evaluation methods was assessed using the Cohen’s kappa test and interpreted according to the criteria of Landis and Koch [22].

A multivariate logistic regression model was used to analyse the risk and confounding factors. All variables that showed significance in the bivariate tests were included in the model (length of stay, number of off-label drugs prescribed per patient, number of drugs prescribed per patient, weight, sex and age).

### Ethical considerations

This study was performed in line with the principles of the Declaration of Helsinki. The approval of the Ethics Committee of Hospital Clínico San Carlos was obtained for its development (Code:18/340-E), and written informed parental consent was obtained for all patients.

## Results

The recruitment period of our study coincided with the outbreak of the COVID-19 pandemic. As a result, scheduled surgeries were cancelled or delayed, thereby reducing the number of recruitable patients. A total of 1699 patients underwent surgery during this period, of which 311 patients (117 girls and 194 boys) were assessed.

The age of patients ranged from 1 to 16 years (7.4 ± 4.6 years; median: 6 years), the most common ages were 4 and 5 years. These common age groups consisted of 32 patients each (10.3% of the cohort).

For 239 (76.8%) patients the length of stay in hospital was less than one day. Hospitalisation lasting more than one day was less frequent. Only one patient (0.3%) remained in hospital for one week (8 days). The average length of stay was 0.38 ± 0.98 days (95% CI 0.28 to 0.49).

Eighty surgeries were performed. The most common interventions included adenoidectomy (45, 12.1%), circumcision (38, 10.2%), and tonsillectomy (30, 8.1%).

All patients received at least one drug during hospitalisation. A total of 2873 drug prescriptions were assessed. The highest number of drugs administered to a single patient was 21. The average number of prescriptions was 9.24 ± 4.18 per patient. The full list of drugs prescribed according to the ATC classification is shown in Table 1.

**Table 1:** Description of the medicine groups prescribed [anatomical-clinical classification (ATC), levels 1 and 2]

ATC CLASSIFICATION	*N*	%
**GROUP A: ALIMENTARY TRACT AND METABOLISM**		
A02: Drugs for acid-related disorders	234	8.14
A04: Antiemetics and antinauseants	282	9.82
**GROUP H: SYSTEMIC HORMONAL PREPARATIONS**		
H02: Corticosteroids for systemic use	232	8.08
**GROUP J: ANTI-INFECTIVES FOR SYSTEMIC USE**		
J01: Antibacterials for systemic use	165	5.74
**GROUP M: MUSCULOSKELETAL SYSTEM**		
M01: Anti-inflammatory and antirheumatic products	178	6.20
M03: Muscle relaxants	95	3.31
**GROUP N: NERVOUS SYSTEM**		
N01: Anaesthetics	721	25.10
N02: Analgesics	806	28.05
N05: Psycholeptics	71	2.47
**OTHER**	89	3,10
**Total**	**2873**	**100%**

Eighty patients (25.7%; 27 girls, 53 boys) experienced at least one ADR, and 244 drug uses resulted in ADRs, representing 8.5% of the 2873 drug prescriptions.

All ADRs occurred during anaesthesia induction or immediately after anaesthesia recovery. No late onset ADRs were observed or notified by the patients after discharge. In total, 13 different types of ADRs were observed (Table 2), of which hypotension (32; 35%), nausea (16; 15.5%), and emergence delirium (16; 15.5%) were the most frequent.

**Table 2:** List of most common Adverse Drug Reactions, clinical manifestations and mechanism

Adverse drug reaction description	Clinical manifestations	Mechanism	*N*	%
Hypotension	Blood pressure < lower limit proposed for the age group, according to the table described in the Harriet Lane handbook.	• Drug-drug interaction (29)	36	35%
		• Increased pharmacological effect (7)		
Nausea	A feeling of sickness or discomfort in the stomach that presents as an urgent need to vomit, without the act of vomiting.	• Drug-drug interaction (15)	16	15.5%
		• Increased pharmacological effect (1)		
Emergence Delirium	Disturbance in a child’s awareness or attention to his/her environment with disorientation and perceptual alterations. This includes hypersensitivity to stimuli and hyperactive motor behaviour in the immediate post anaesthesia period.	• Idiosyncrasy (16)	16	15.5%
Vomiting	Act of emptying the contents of the stomach through the mouth (bilious or food content).	• Drug-drug interaction (11)	12	11.7%
		• Increased pharmacological effect (1)		
Tachycardia	Heart Rate > upper limit proposed for the age group, according to the table described in the Harriet Lane handbook.	• Drug-drug interaction (4)	5	4.9%
		• Increased pharmacological effect (1)		
Oxygen desaturation	Reduction in oxygen saturation < 94% without other systemic symptoms.	• Drug-drug interaction (3)	4	3.9%
		• Increased pharmacological effect (1)		
Prolonged neuromuscular blockade	Duration of the neuromuscular blocker effect is longer than the required time for the procedure; therefore, reversal administration such as sugammadex is required. This includes patient metabolic characteristics that can cause prolonged effects and overdosing of the blockers.	• High level of Doses (3)	3	2.9%
Bradycardia	Heart Rate < lower limit proposed for the age group, according to the table described in the Harriet Lane handbook.	• Drug-drug interaction (1)	3	2.9%
		• Increased pharmacological effect (2)		
Dizziness	Subjective feeling of being dizzy, floating, or surrounding objects spinning.	• Drug-drug interaction (2)	2	1.9%
Drowsiness	After the procedure, until discharge, the patient spends most of the time asleep or frequently feels the need to sleep again.	• Drug-drug interaction (1)	2	1.9%
		• Increased pharmacological effect (1)		
Acute respiratory depression	Reduction in oxygen saturation < 92% combined with other clinical symptoms such as hypotension, dyspnea, or use of accessory muscles, which requires the administration of oxygen.	• Drug-drug Interaction (2)	2	1.9%
Bronchospasm	Shortness of breath associated with wheezing, pain, or tightness in the chest and back.	• Increased pharmacological effect (1)	1	1%
Headache	Pain or discomfort in head or face area.	• Other: the event occurred because of the administration technique (subconjunctival injection). (1)	1	1%
**Total**			**103**	**100%**

Of the ADRs identified, 87 (84.5%) were expected reactions according to the drug’s mechanism of action (Type A), and 16 (15.5%) were unexpected idiosyncratic drug reactions (Type B). Most of these events occurred because of drug interactions (synergism, additivity, or potentiation). The drug combination more frequently responsible for these events was sevoflurane and fentanyl [32 of the ADRs (31.1%)]. A combination of these two drugs and propofol, caused 13 of the ADRs (12.6%). Finally, the addition of dipyrone to the last combination (sevoflurane + fentanyl + propofol), caused 10 (9.7%) of all ADRs.

For 44 (42.7%) ADRs, no action was taken because the ADRs were identified retrospectively by the investigators after reviewing the clinical records, and/or no measures were required to reverse the reaction. In 58 (56.3%) patients, treatment to counteract the ADR was carried out, and in one case (1%) the suspected drug was discontinued.

Concerning causality, the same number of ADRs were detected by both algorithms. According to the Naranjo method, 84 (81.8%) ADRs were considered “probable”, 11 (19.7%) “possible”, and 8 (7.8%) “definitive”. Upon the Karch-Lasagna algorithm, 78 (75.7%) were classified as “probable”, 15 (14.6%) “possible”, and 10 (9.7%) “defined”. Moreover, the concordance evaluation results displayed a Cohen’s Kappa statistic of 0.73 (κ = 0.73). This value is categorized as “substantial” according to Landis and Koch’s criteria.

According to the Venulet criteria, 97 (94.2%) ADRs were considered mild, two (1.9%) moderate, and four (3.9%) severe. Similarly, when the ADRs intensity was assessed according to Spanish Pharmacovigilance System criteria, four (3.9%) cases were considered serious and 99 (96.1%) non-serious. Three of the four ADRs that were considered serious by both algorithms occurred in the respiratory system (bronchospasm and acute respiratory depression) and one in the nervous system (prolonged neuromuscular blockade).

Concerning the potential avoidability of the ADRs, most cases were considered “Not avoidable” (*N* = 96; 93.2%), and 7 (6,8%) ADRs were considered “Possibly avoidable”.

Using the bivariate model, the probability of a prescription causing ADR was significantly higher when off-label vs. labeled prescriptions were used [X^2^ = 762.13; *p* < 0.001; OR = 62.32; 95% CI 38.85 to 99.99] (Table 3).

**Table 3:** Contingency table of labelled vs. off-label drug prescriptions in the development of ADRs

		Adverse Drug Reaction (ADR)		Total
		Yes	No	
**Off-label drug prescription**	**Yes**	57 (55,3%)	54 (1,9%)	111 (3,9%)
	**No**	46 (44,7%)	2716 (98,1%)	2762 (96,1%)
**Total**		103 (100%)	2770 (100%)	2873 (100%)

X2 = 762,13; *p* < 0,001; OR = 62,324; 95% CI 38,847 to 99,988

Similarly, the probability of a patient suffering from at least one ADR was significantly higher when using off-label vs. labelled drugs (X^2^ = 33.304; *p* < 0.001; OR = 4,674; 95% CI 2.71 to 8.06) (Table 4).

**Table 4:** Contingence table of patients with off-label prescriptions and ADR

		Adverse Drug Reaction (ADR)		Total
		No	Yes	
**Off-label drug prescription**	**No**	185 (80.1%)	37 (46.3%)	222 (71.4%)
	**Yes**	46 (19.9%)	43 (53.8%)	89 (28.6%)
**Total**		231 (100%)	80 (100%)	311 (100%)

**Fig. 1:**
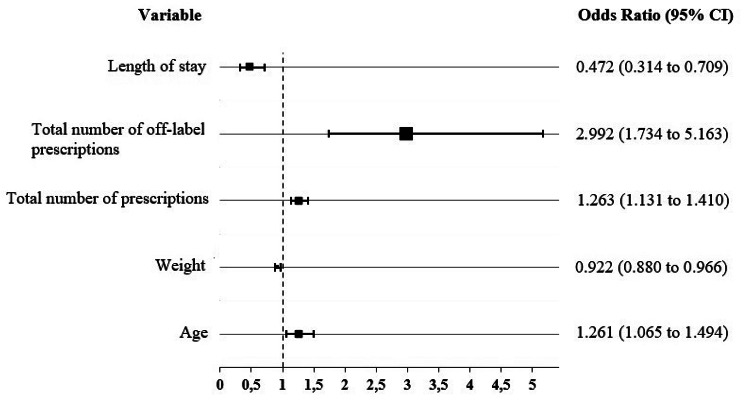
Forest plot on the results of multivariate logistic regression analysis of risk factors associated with ADR development

X^2^ = 33.304; *p* < 0.001; OR = 4,674; 95% CI 2.71 to 8.06,

Using the logistic regression model [See additional File 1], all variables were significant, except for sex (*p* = 0,815). The number of off-label drugs prescribed per patient was the variable most strongly associated with the development of ADRs (OR = 2.99; 95% CI 1.73 to 5.16), followed by the number of drugs prescribed per patient (OR = 1.26, 95% CI 1.13 to 1.41), and age (OR = 1.26, 95% CI 1.07 to 1.49). On the contrary, a relatively protective effect is shown for the length of hospital stay (OR = 0,472, 95% CI 0.134 to 0.709) and a very little for the weight (OR = 0,922, 95% CI 0.080 to 0.966). (Fig. 1).

## Discussion

The COVID-19 pandemic impacted our study by decreasing scheduled surgeries and therefore delaying patient recruitment, though no differences, upon clinical impression, in patient characteristics or interventions were observed before and during the pandemic.

All the anaesthesiologists and surgeons who participated in the surgeries involved in this study had specific training and at least 5 years of experience in the area of paediatrics.

The anaesthesia protocols and standards relied in the use of balanced anaesthesia technique, which included an inhaled anaesthetic agent, a hypnotic drug, an opioid agent, and in most cases added a neuromuscular blocker, following the recommendations from one of the most relevant manuals of Anaesthesia [26]. These practices are considered similar to any other hospital.

Regarding the incidence, a relatively high ADR frequency was identified in our study, as 25.7% of the patients experienced at least one ADR during the assessment. Although no publication was found on the frequency and characteristics of ADRs in the paediatric surgical population (during surgery, post-surgery, or hospitalisation periods), the incidence detected in our study was similar to that found by other authors [21, 23, 24, 25] in paediatric ICUs, where the drugs used could be considered quite similar to those administered to our patients.

Many authors have reported ADRs in the gastrointestinal system as the most frequent [9, 27, 28, 29] while in our study it is the vascular system (specifically hypotension) the most frequently affected, placing gastrointestinal ADRs in second place. This agrees with the findings of a publication [10] on ADRs frequency in the surgical general population, in which 3.1% of the patients were paediatric. On the other hand, when we consider the literature that analyses the same setting in the adult population in a surgical ICU [26], it also identified gastrointestinal and those affecting the vascular system as the most frequent ADRs.

Regarding the methods of causality assessment, the Cohen’s kappa value (κ = 0.73) indicated that the two methods used (Naranjo [16] and Karch-Lasagna [17]) treated cases similarly. Both methods have been widely used in the assessment of paediatric ADRs, without requiring validation for this age group, as the elements they evaluate (temporal sequence, prior knowledge of ADRs, etc.) don’t differ from adults to children. However, an algorithm assessing causality in the anaesthetic setting is needed, because of the frequent concomitant administration of multiple drugs in the surgical environment.

Most of possibly avoidable ADRs are associated with the administration of sevoflurane alone or in combination with other drugs. This was discussed with the patient’s clinicians who were aware of the past ADRs related to medication, even though, in general, the re-administration of the drug in these patients, was considered adequate in terms of the benefit-risk ratio as other alternatives may have more severely impacted the child. (e.g. the occurrence of hypotension when combining sevoflurane with propofol, in low doses, in favor of better airway management). On the other hand, the high percentage of administered corticosteroids might lead to suspect the existence of immune-related ADRs. However, in this case it corresponds to intraoperative dexamethasone administration for prevention of postoperative nausea and vomiting (PONV).

Regarding the factors related to risk of ADRs, an increase in the length of stay at hospitals has often been associated with a higher probability of identifying ADRs [29, 30]. This was not the case in our study (OR = 0.47; 95% CI 0.31 to 0.71); which could be affected by the fact that most of the patients were hospitalized for less than one day. Also, lower age groups have been commonly associated with a higher risk of ADRs [31, 32], while we observed the opposite association in our study (OR = 1.26; 95% CI 1.07 to 1.49). This may be due, on one hand, to the fact that more complex surgical procedures occurred in older children who required a greater number of medications, and on the other hand, to the fact that their communication skills are more developed than in younger children. Therefore, they more frequently verbalize symptoms that allowed us to identify ADRs.

Consistently with other studies [5, 33–35], off-label prescriptions of commercialized medicines for children have been identified as a cause of ADRs. In our study, the risk was more than 60-fold of that of the labelled prescriptions, and after correcting for confounding factors using the regression model, a significant 3-fold risk was observed. These results seem reasonable considering the physiological differences [36] between adults (in whom most of the drugs have been studied) and children, and the fact that the safety profile of these medicines has not been tested in paediatric clinical trials.

Some well-authorized persons managing paediatric prescriptions, such as the specialists members of the Spanish Association of Paediatrics [37], consider the off-label use of medicines in paediatrics as necessary. The absence of specific formulations for this population often places them in a position of therapeutic orphanhood, so that the benefit-risk balance tilts towards the use of these products, highlighting the need for trials in this population.

## Conclusion

In conclusion, ADRs had a high incidence rate in our paediatric surgical cohort, and the off-label use of drugs was a key risk factor for ADR development.

### Electronic supplementary material

Below is the link to the electronic supplementary material.

Supplementary Material 1

## Data Availability

Data may be obtained from the author upon request.

## Abbreviations

ADR: Adverse Drug Reaction
AE: Adverse Events
ADE: Adverse Drug Events
ATC: Anatomical Therapeutic Classification
CI: Confidence Interval
PONV: Postoperative nausea and vomiting
SEFV-H: Spanish Pharmacovigilance System for Medicinal Products for Human Use
SD: Standard Deviation
SPSS: Statistical Package for the Social Sciences
OR: Odds-ratio
P: *p*-value
WHO: World Health Organization
X^2^: Chi-Squared Test
