# Supplementary material for: Adverse drug reactions in paediatric surgery: prospective study on frequency and risk related factors
Source: BMC Pediatr. 2024 May 18;24:344. doi: 10.1186/s12887-024-04803-1 (PMC11102197; doi:10.1186/s12887-024-04803-1)
Supplement: Supplementary file 1 — Supplementary Material 1 [file 12887_2024_4803_MOESM1_ESM.docx]

| **Additional File 1:** ***Multivariate logistic regression analysis of risk factors associated with Adverse Drug Reactions development.*** | | | | | |
| --- | --- | --- | --- | --- | --- |
| **Variable** | **B** | **Sig.** | **EXP B (Odds-ratio)** | **95% CI** | |
|  |  |  |  | **Lower limit** | **Upper Limit** |
| **Sex** | 0,067 | 0,815 | 1,070 | 0,575 | 1,992 |
| **Age** | 0,232 | 0,008 | 1,261 | 1,065 | 1,494 |
| **Weight** | -0,081 | 0,001 | 0,922 | 0,880 | 0,966 |
| **Total number of prescriptions** | 0,233 | 0,000 | 1,263 | 1,131 | 1,410 |
| **Total number of off-label prescriptions** | 1,096 | 0,000 | 2,992 | 1,734 | 5,153 |
| **Length of stay** | -0,751 | 0,000 | 0,472 | 0,314 | 0,709 |
| **Constant** | -2,845 | 0,000 | 0,058 |  | |
